# gViz, a novel tool for the visualization of co-expression networks

**DOI:** 10.1186/1756-0500-4-452

**Published:** 2011-10-27

**Authors:** Raphaël Helaers, Eric Bareke, Bertrand De Meulder, Michael Pierre, Sophie Depiereux, Naji Habra, Eric Depiereux

**Affiliations:** 1 Bioinformatics and Biostatistics unit, Molecular Biology Research Unit (MBRU), Namur Center for Complex Systems (NAXYS), University of Namur (FUNDP), 61 Rue de Bruxelles, B-5000 Namur, Belgium; 2 Research Center in Information Systems Engineering (PReCISE), Faculty of Computing, University of Namur (FUNDP), 21 Rue Grandgagnage, B-5000 Namur, Belgium; 3 Laboratory of Human Molecular Genetics (GEHU), de Duve Institute, Catholic University of Louvain (UCLouvain), 75 Av. Hippocrate, B-1200 Brussels, Belgium

## Abstract

**Background:**

The quantity of microarray data available on the Internet has grown dramatically over the past years and now represents millions of Euros worth of underused information. One way to use this data is through co-expression analysis. To avoid a certain amount of bias, such data must often be analyzed at the genome scale, for example by network representation. The identification of co-expression networks is an important means to unravel gene to gene interactions and the underlying functional relationship between them. However, it is very difficult to explore and analyze a network of such dimensions. Several programs (Cytoscape, yEd) have already been developed for network analysis; however, to our knowledge, there are no available GraphML compatible programs.

**Findings:**

We designed and developed gViz, a GraphML network visualization and exploration tool. gViz is built on clustering coefficient-based algorithms and is a novel tool to visualize and manipulate networks of co-expression interactions among a selection of probesets (each representing a single gene or transcript), based on a set of microarray co-expression data stored as an adjacency matrix.

**Conclusions:**

We present here gViz, a software tool designed to visualize and explore large GraphML networks, combining network theory, biological annotation data, microarray data analysis and advanced graphical features.

## Background

The merging of network theories, public biological data knowledge and microarray data analysis techniques presents an unexploited opportunity to explore and understand the functionality of genes. Similar patterns in gene expression profiles have been assumed to suggest relationships between genes [1] and it is important to discover these relationships between co-expressed genes using co-expression matrices from microarray data. While the construction of co-expression networks may be straightforward, we limit our work to the presentation of a visualization tool to search for co-expression between genes and leave to other studies the important question of determining whether it is biologically meaningful to represent a gene by a network node and a functional relationship by an edge.

Several network exploration softwares have been developed these past years; each of them with particular pros and cons. We direct readers to the following reviews for more details on those programs [2,3]. After testing some of the programs discussed in those reviews, we estimated there was an advantage to build our own visualization program, with several new features included.

Our motivations for developing our program were to allow use of the GraphML (Graph Markup Language) format into a network visualization software and providing a biologists-oriented network exploration solution, both user-friendly and light. To the best of our knowledge, gViz is the only software capable of rendering dynamically GraphML networks. We are aware of the Cytoscape plugin 'Graphmlreader', however it is still in development and seems to be non-functioning at the moment.

The GraphML format is based on XML structure and is therefore ideally suited as a common denominator for all kinds of services generating, archiving, or processing graphs. It supports attributed for nodes and edges, hierarchical graphs and is very flexible [4]. GraphML was developed as modern graph exchange format, suitable in particular for exchange between graph drawing tool and other applications, during the 8^th ^Symposium on graph drawing (GD 2000). It has been developed with the following pragmatic goals in mind: simplicity, generality, extensibility and robustness [5]. We also noticed that this format is very compact and therefore allows for faster transfer or loading into a software. One given network uses less storage space when encoded in GraphML format then, for example, in DOT format.

gViz is a novel tool built around clustering coefficient-based algorithms to visualize and manipulate networks of co-expression interactions among a selection of probesets (each probeset representing a single gene or transcript), based on a set of microarray co-expression data stored as an adjacency matrix. This adjacency matrix, representing numerically the relationships between probesets, can be inferred using co-expression data computational tools such as MINET [6], an R package that computes co-expression relations between probesets, and several microarrays.

The inference of interaction networks is a relatively new area in the field of bioinformatics. However, several algorithms are available, including but not limited to the following: Bayesian approach-based algorithms, such as the GeneTS package [7], neuronal computation model algorithms, such as the "neuro-fuzzy" [8] approach, or mutual information computation-based algorithms, such as MINET or qpGraph [9]. We chose to use MINET for several reasons: at each step of the computation process, it is possible to choose between several methods and to set different parameters; the methods contained in the algorithm are at the cutting edge of current knowledge in the field; and finally we were able to interact with the developers of the package to refine certain steps of the computation. MINET accepts preprocessed microarray data (we chose to use the GCRMA [10] preprocessing method) and provides a co-expression matrix in the form of a GraphML file.

Even if input co-expression matrices are based on probeset identifiers, gViz provides users with advanced features to visualize the corresponding interaction networks using other associated identifiers, such as gene identifiers (Entrez Gene IDs [11], Ensembl IDs [12], UniGene IDs [13] and gene symbols), protein identifiers (SwissProt Accession Codes [14]) and disease identifiers (OMIM IDs [15]).

## Implementation

gViz is written in Java and uses JUNG 2 (Java Universal Network/Graph Framework, http://jung.sourceforge.net/), a Java library that provides a common and extensible language for the modeling, analysis and visualization of data that can be represented as a graph or a network. Annotation information displayed on the graph originates from an external microarray database, PathEx [16], a manually-curated web-based expression array dataset builder of human microarray data, for researchers who need to generate organized microarray datasets efficiently and according to their specific needs.

## Results and discussion

### Comparison with existing software

**Figure 1:**
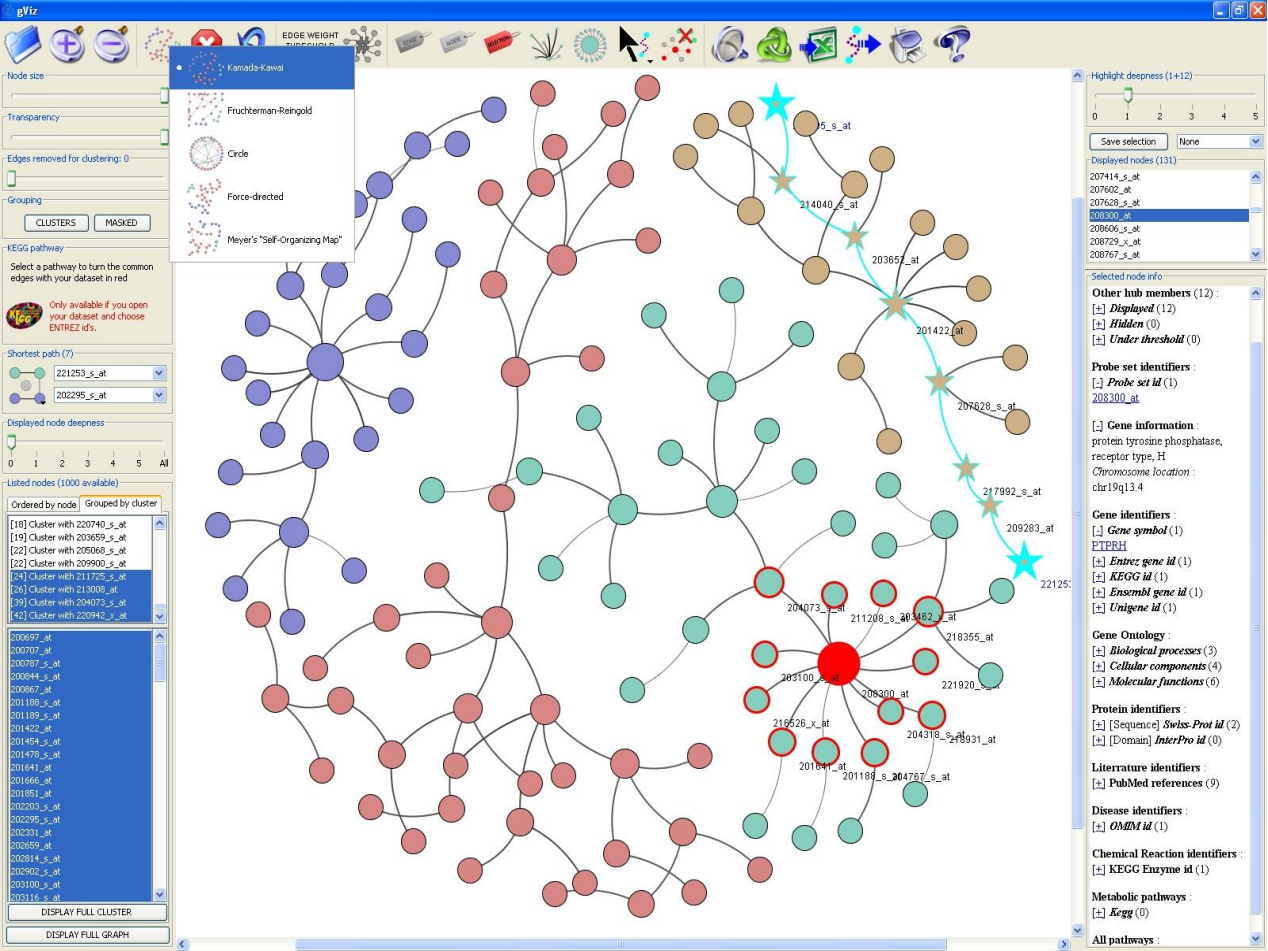
**Screenshot of the general interface of gViz**. This is the interface of a typical analysis in gViz. The center panel displays the current network. The top panel contains the visual functionalities. The left panel contains the different sliders as well as the clustering and comparison tools. Finally, the right panel shows the information contained in PathEx on the nodes selected in the current network.

**Figure 2:**
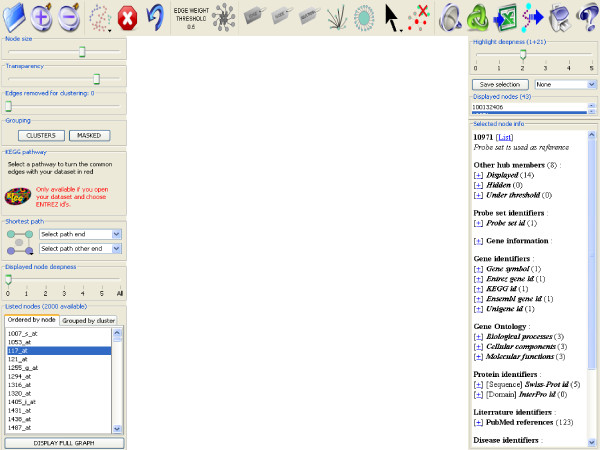
**Zoom of the top, left and right panels**. Detailed view of the top panel where are grouped the visual tools of gViz, left panel containing the sliders along with the clustering and comparison tools and right panel presenting the information from PathEx on the selected genes, as well as slider to adjust the selection deepness.

**Figure 3:**
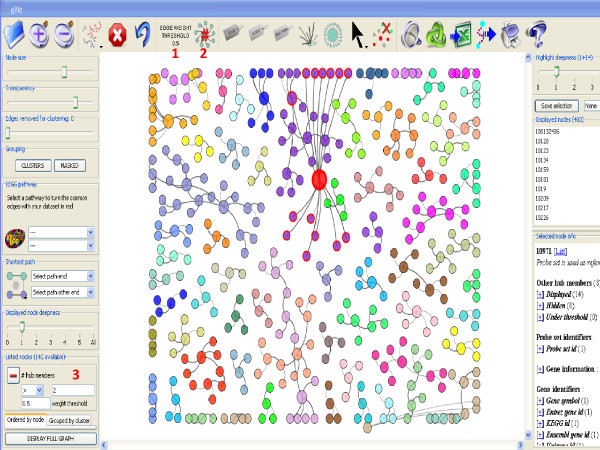
**Filters in gViz**. This figure displays a network filtered by the number of neighbors (> 2) and the Minet score (score > 0.5). The filters are triggered by the buttons in {1} and {2}, and controls for the filters are accessed in the left lower panel {3}.

Several programs have been designed for the analysis of biological networks, the best known of which is Cytoscape [17]. Although it is a major first class visualization program, we chose to develop gViz and to add some unique and convenient functionalities, the first of which is the capacity to compute and display sub-networks using several different built-in filters (see Figure 1 and 2 for an overview of gViz interface and functionalities). This functionality, although potentially biologically important, does not have its counterpart in Cytoscape (see Figure 3). We also noted that gViz is much more user-friendly and easier to use than most of the alternative software packages. Although it is technically possible to display a huge network containing tens of thousands of nodes and a hundred times more edges, it would not be humanly efficient to identify specific parts of that network. In that context, gViz proposes various features that allow the user to display only parts of the network of interest. The user can then select one or more identifiers from a list deriving from a data set provided and display the sub-network containing only the relationships to be studied. A "deepness" slider feature provided can be used to gradually adjust the neighborhood of the selected identifiers: if n identifiers are selected and a deepness of d is chosen, the sub-network displayed will include the n identifiers and all their neighbors at a distance of a maximum of d edges. For example, selecting a single node and a deepness of 2 will display the selected node, its immediate neighbors and neighbors of the immediate neighbors. It is also possible to set deepness to the "maximum", hence displaying all neighbors reachable from the selected identifiers, regardless of their distance.

gViz can also filter the displayed relationships (edges) by excluding those with a weight under a given threshold (determined during the computational part in MINET; the weight represents the certainty of the selected interaction; i.e., if we consider a pair of nodes i and j, the weight of the arc between them is the maximum of the MRMR (maximum relevance/minimum redundancy) score computed in both directions.). The user can at any time reduce (or increase) the number of edges displayed by eliminating those which are most likely to be false positives.

Apart from filtering by edge weight, one can filter the node list by degree (i.e. number of neighbors) and/or by clusters (i.e. sub-networks without a connection between them). The clusters are recalculated whenever the user changes the threshold of the edge weights, because they do not take into account edges excluded by the filter. Another prominent feature allows the user to find identifiers by providing annotation criteria (such as a description of a gene or its involvement in a biological process), and generate a sub-network using all or part of the search results.

It is worth mentioning that, to our knowledge, gViz is one of the few software packages capable of displaying and analyzing GraphML-based networks at the genome scale. The yEd graph editor [18] is another piece of software able to display GraphML; however, it is less powerful and proposes fewer features than gViz. The GraphML format was introduced around the year 2000 as a common network information exchange format. As such, gViz is a serious candidate for widespread GraphML analysis.

Here is a summary of gViz main advantages: an important feature of gViz is the ability to render dynamic networks. This feature does not appear in most network visualization software (Rhodobase, Starnet, ... [19,20]), although it does in Cytoscape. gViz has also a unique additional feature of reading and displaying GraphML-based networks. Another important feature of gViz is the possibility to filter networks on the basis of different criteria, topological (number of neighbors, strength of edges, ...) and biological ones (membership in a certain pathway, known interactions, ...). The filtering ability based on several criteria does appear in other programs; however the set of criteria on which one can filter networks in gViz is unique. We believe that some of the filters available in gViz will be very useful to biologists.

### gViz functionalities

Networks displayed by gViz are dynamic and can be manipulated; each node or group of nodes can be moved using the mouse. Several layout algorithms are available to automatically position graph nodes and the user can hence organize nodes differently depending on the complexity of the actual network. Other 'visual' features include the possibility to change the size and transparency of nodes, to set edge thickness as a function of their weight, or to set node diameter as a function of their degree. Specific nodes can be selected directly on the network graph using the mouse or from a list of displayed identifiers, and their neighbors are highlighted (the number of neighbors is set using a desired distance). Users can also generate colored clusters differently by choosing the number of edges to remove to form highly connected sub-networks, where these edges are identified using the Girvan and Newman clustering algorithm [21].

When one or more nodes of the network are selected, gViz displays a range of annotation information from the displayed sub-network, such as the list of direct neighbors (displayed or not in the sub-networks) and the neighbors reached by an edge below the threshold set for the edge weight, and from a biological database, such as the probeset identifier, information on associated genes, biological processes, cellular components and molecular functions involved (from Gene Ontology [22]), proteins (sequences from SwissProt and domains from InterPro [23]), references in the literature (from PubMed [24]), diseases in which corresponding genes are involved (from OMIM), chemical reactions and pathways in which these genes are involved (from KEGG [25] and GenMAPP [26]). gViz also has two features that display the shortest path between two nodes and highlight the edges of the sub-network that can be identified in a pre-selected KEGG pathway. Various statistics on the sub-network can be obtained, including histogram plots providing the user with the degree of network node distribution, the diameter of the sub-network, the weight distribution of the edges, the graph distribution coefficient of clustering [27] and/or the cluster size distribution. Finally, the displayed (sub-)network and its statistics can be exported into different image and data file formats.

### Case study

In this section, we will give an example of how to collect data, generate a GraphML using Minet and R and explore the resulting graph in gViz.

- Data collection: There are several web repositories for microarray data (GEO [28], Array Express [29]). We recommend the use of our database PathEx, for easier and faster data collection.

- Network Computation:

We use the following packages to compute our networks: GCRMA, Minet and Infotheo (all of which freely available for download in Bioconductor).

Here follows the R code to compute the network from microarray.cel files (for R > = R2.10)

library(gcrma)

library(minet)

cel < -list.celfiles()

a < -ReadAffy(filenames = cel)

b < -gcrma(a)

c < -exprs(b)

d < -t(c)

disc < -discretize(d, disc = "equalfreq ", nbins = sqrt(nrow(d)))

mim < -mutinformation(disc)

net < -mrnet(mim)

write.table(net, file = "net.txt ", sep = " \t")

Once these steps are done (which can be long, depending on the computer's speed and dataset size), one can import the network computed into gViz.

- gViz exploration

**Figure 4:**
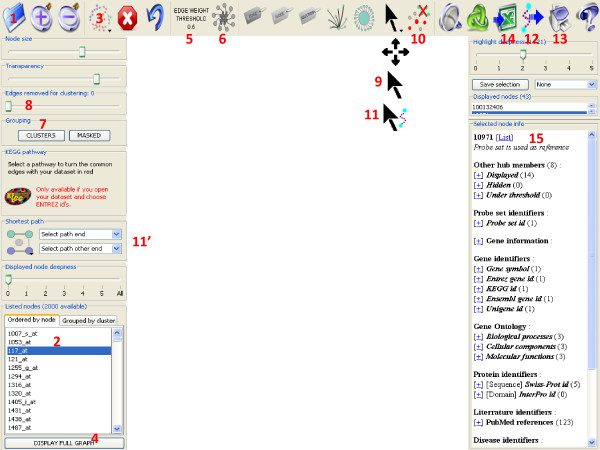
**Case study in gViz**. Detailed view of the gViz interface and location of the tools mentioned in the 'case study' section. Legend: 1-Open button; 2- Lower left panel, list of nodes available; 3- Layout control button; 4- Display full graph button; 5- Filter on edges scores; 6- Filter on number of neighbors; 7- Clustering button; 8- Edges removed for clustering control slider; 9- Selection tool; 10- Remove non-selected nodes buttons; 11- Shortest path tool; 12- Export shortest path button; 13- Export network (png); 14- Export network (text); 15- Lower right panel, information on selected nodes.

Once the network is loaded in gViz (using the 'open' button {1}, see Figure 4 and the gViz manual), the list of the available nodes (genes) is shown in the lower left panel {2}. To display the entire graph, first select the 'circle' layout {3} then click on 'display full graph'{4}. The circle layout is recommended for its low computational needs; rendering a very large network can be extremely resource consuming. However, this step allows for a first overview of the network. At this step, one might want to filter his graph, using the filter on the Minet score {5}, or the filter on the number of neighbors{6}. One can also use the clustering option {7} to group similar nodes, by removing progressively the weakest edges in the graph (controlled by the 'edge removed for clustering' slider {8}). Then, using the selection tool {9}, one highlights the nodes of interest, then hits the 'remove non-selected nodes' button {10}. The resulting sub-graph can then be shown using another, more explicit, layout (for example, 'force-directed').

The different layouts available in gViz have different uses: the 'circle' layout suits best the very big graphs, as it requires less computational power to be displayed. When working on a mid-sized sub-graph (less than 1,000 nodes), the 'Kamada-Kawai' or 'Fruchterman Reingold' layouts are suggested. 'Force directed' or 'Meyer's self organizing' layouts are best suited for small (less than 100 nodes) networks, as they need more computational power to work but better discriminate the edges.

To compute the shortest path between two nodes, first select the 'shortest path' tool {11}, click on the first node of interest then maintain the SHIFT key and click on the second node. gViz automatically computes the shortest path between those two nodes, with respect to the filters possibly applied. This feature can also be controlled via the left panel {11'}. The shortest path can be exported using the 'Export shortest path' button {12}.

It is possible to export the current graph in image (click on 'export network (png)' button {12}) or in various text formats (using the 'export network (text)' button {13}).

To obtain information on a certain node, simply click on its name in the lower right panel {14}, displaying all the information contained in the PathEx database for this particular gene (for more information about PathEx, see [16]).

## Conclusions

This manuscript presents gViz, a new software package for the visualization, exploration and analysis of GraphML-based genetic co-expression networks. It has many convenient built-in filtering and displaying features and is connected to an external database containing gene and annotation information. In conjunction with the MINET R package, we use gViz to explore the co-expression relationships of genes in different cellular states.

## Availability and requirements

gViz is available at http://urbm-cluster.urbm.fundp.ac.be/webapps/gviz for 32 and 64 bit Windows, MacOS × and Linux/Unix. It requires Java engine 1.6 or higher to run http://java.com. To connect with the external database PathEx, the port 3306 of the user's computer must be opened. This port may be blocked by a firewall and thus users must ask their network administrator to unblock it for their machine, if necessary. gViz is available under the Open GPL license.

## Competing interests

The authors declare that they have no competing interests.

## Authors' contributions

RH performed the major part of the coding, as well as debugging and web-publishing of the software. EB developed the PathEx database and took part in the coding and debugging process. BDM took part in the design, debugging and biological testing. MP and SD participated in the biological testing process. NH was involved in the design and implementation process. ED conceived the study and supervised the development and publication. All authors have read and approved the final manuscript.
